# Accuracy of the Arabic HCL - 32 and MDQ in detecting patients with bipolar disorder

**DOI:** 10.1186/s12888-023-04529-x

**Published:** 2023-01-26

**Authors:** Uta Ouali, Yosra Zgueb, Lamia Jouini, Amina Aissa, Rabaa Jomli, Abdelhafidh Ouertani, Adel Omrani, Fethi Nacef, Mauro G. Carta, Antonio Preti

**Affiliations:** 1 Department Psychiatry A, Razi Hospital, Rue des Orangers, 2010 La Manouba, Tunisia; 2 Faculty of Medicine of Tunis, University of Tunis El Manar, Tunis, Tunisia; 3 Research Laboratory LR18SP03, Tunis, Tunisia; 4 Centre de Compétences en Psychiatrie et Psychothérapie, Pôle de Psychiatrie et Psychothérapie, Hôpital du Valais (HVS)- Centre Hospitalier du Valais Romand, Sion, Switzerland; 5 Tunisian Bipolar Forum, Erable Médical Cabinet 324, Tunis, Tunisia; 6 Department of Medical Sciences and Public Health, University of Cagliari, Cagliari, Italy; 7 Department of Neuroscience, University of Turin, Turin, Italy

**Keywords:** Bipolar disorder, Depression, Screening, Hypomania check list (HCL - 32), Mood disorder questionnaire (MDQ), Hypomania

## Abstract

**Background:**

Studies about the two most used and validated instruments for the early detection of Bipolar Disorder (BD), the 32 - item Hypomania Checklist (HCL - 32) and the Mood Disorder Questionnaire (MDQ), are scarce in non-Western countries. This study aimed to explore the reliability, factor structure, and criterion validity of their Arabic versions in a sample of Tunisian patients diagnosed with mood disorders.

**Methods:**

The sample included 59 patients with BD, 86 with unipolar Major Depressive Disorder (MDD) and 281 controls. Confirmatory factor analysis was applied to show that a single global score was an appropriate summary measure of the screeners in the sample. Receiver Operating Characteristic analysis was used to assess the capacity of the translated screeners to distinguish patients with BD from those with MDD and controls.

**Results:**

Reliability was good for both tools in all samples. The bifactor implementation of the most reported two-factor model had the best fit for both screeners. Both were able to distinguish patients diagnosed with BD from putatively healthy controls, and equally able to distinguish patients diagnosed with BD from patients with MDD.

**Conclusion:**

Both screeners work best in excluding the presence of BD in patients with MDD, which is an advantage in deciding whether or not to prescribe an antidepressant.

**Supplementary Information:**

The online version contains supplementary material available at 10.1186/s12888-023-04529-x.

## Background

Bipolar disorder (BD) is a severe mental disorder with a chronic-recurring course. Since the first episode of a BD is often of a depressive kind [1], BD is often misdiagnosed as major depressive disorder (MDD) [2, 3] and treated as such. This may lead to adverse consequences, such as increased suicide risk [4, 5], greater probability of hospitalization [5], poorer response to antidepressants, and antidepressant-induced switch to mania [6]. Because of its course characterized by recurring episodes separated by periods of euthymia with no or scant symptoms of hypomania, BD may persist undiagnosed for a long time unless a frank episode of mania erupts [7, 8]. Minor hypomanic episodes are often overlooked, and, indeed, the differentiation of clinically elated and irritable mood or increased activity from “normal” variation in the population is often challenging. On average, the duration of undiagnosed, hence untreated, BD may last up to 10 years, and there is some evidence that up to one-third of patients with BD are misdiagnosed at least once during their lifetime [9, 10].

Early identification of BD is essential for appropriate treatment [11]. Several self-report tools have been developed to identify people with possible or probable BD [12]. Self-report screening tools are brief and cost-effective and can be preferred in the busy clinical setting to standardized interviews, which are more accurate but are time-consuming and require appropriate training for the administration and scoring. Nevertheless, caution should be applied in deriving epidemiologic estimates from case-finding based on screening tools [13]. Two of the most used and validated instruments for the early detection of BD are the 32-item Hypomania Checklist (HCL - 32) [11], and the Mood Disorder Questionnaire (MDQ) [14]. These two instruments have been validated in many countries [11, 15–24]. There is evidence that both the HCL - 32 and the MDQ have acceptable psychometric properties and appear to be useful screening tools for BD [25].

Most studies on the HCL - 32 and the MDQ have been carried out in Western countries. The epidemiology of BD shows minor variations by country and ethnicity [26, 27], and the disorder has a likely genetic basis rooted in evolutionary mechanisms [28, 29]. However, cultural factors may influence how symptoms leading to the diagnosis of BD are evaluated [26, 30]. For example, geographical variations in the prevalence of BD might be in part a reflection of the relevance given to the occurrence of psychotic features in BD. The diagnosis of schizophrenia is given priority when the possibility that psychotic features may also occur in the course of BD is overlooked [31]. More subtle influences are related to cultural variations in the patients’ attitudes towards their symptoms. For example, there is evidence from factor analysis that greater involvement in sexual activity, an oft-observed correlate of hypomania, is perceived as a favorable trait by Latin-Mediterranean patients while Asian patients attribute a negative value to hypersexuality, which they tend to associate with other risky behaviors, such as excessive spending or getting in troubles [32, 33]. Studies about the MDQ and the HCL - 32 in non-Western countries are scarce, and most of them are from Asian countries. So far, two studies had explored the reliability and the factor structure of the HCL – 32 [34] and the MDQ [35], respectively, in Arabic-speaking countries. This study aimed at further exploring the reliability, factor structure, and criterion validity of the Arabic version of the HCL - 32 and MDQ in a sample of Tunisian patients diagnosed with a mood disorder (either MDD or BD) by comparison with a sample of putatively healthy people drawn from the general population of Tunisia.

## Material and methods

The study complies with the guidelines of the 1995 Declaration of Helsinki and its revisions [36]. Approval to the study protocol has been granted by the Institutional Review Board (IRB) of Razi Hospital, Tunis, with the authorization signed on 8 Oct 2014.

### Participants

The study was conducted between February 2015 and September 2019 and included a patient group and a control group.

#### Patient group

All consecutive individuals who consulted for the first time at the Department of Psychiatry A of Razi Hospital La Manouba, Tunisia, for signs and symptoms of depression were invited to take part in the study. Individuals were included when the clinician formulated a diagnosis of a current major depressive episode. Thereafter, the Mood Disorder Section of the Tunisian Arabic adapted version of the Structured Clinical Interview for DSM-IV-TR (SCID) was administered by one single researcher (UO) to confirm the diagnosis of Major Depressive Episode and to ascribe the episode to a unipolar or bipolar mood disorder. Resulting diagnosis was (BD) I or II in case a past manic or hypomanic episode were identified, and Major Depressive Disorder (MDD) in case no past manic or hypomanic episode were identified. Additional inclusion criteria were: aged between 18 and 65 years old; having the capacity of providing informed consent. Exclusion criteria were: illiteracy or other cause of inability to read; documented history of mental retardation; and cognitive decline.

#### Healthy control group

Healthy control subjects were included from the general population upon completion of patient recruitment. Control subjects were gender- and age-matched. Inclusion criteria were the absence of a personal history of any psychiatric disorder or consultation in psychiatry, and the absence of a family history of psychiatric disorder in a first-degree relative. In addition, subjects had to answer “no” to both “A” criteria questions for a lifetime major depressive episode of the SCID.

After inclusion and the administration of the SCID in the patient group, subjects of both groups filled out the MDQ and the HCL - 32. All included subjects provided written informed consent.

### Measures

The Arabic version of the MDQ has been used [35]. The MDQ is a self-report tool aimed at screening for potential lifetime indicators of a manic or hypomanic syndrome. It consists of 13 yes/no items evaluating manic symptoms according to DSM-IV criteria [14]. A cut-off of 7 out of 13 items is optimal, in terms of sensitivity and specificity, for identifying BD against healthy people or patients diagnosed with MDD [25].

A Tunisian Arabic version of the HCL - 32 has been used, which was prepared according to standard procedures [37]. At the time of the planning of the study, there was no Arabic version of the HCL - 32. An Arabic version of the HCL - 32 has been published thereafter only [34]. Moreover, each Arabic country has its own dialect, and although there is a standard Arabic language, many people grasp the concepts better in their local Arabic language. As the HCL - 32 has quite a few items which are culturally sensitive (and could therefore be interpreted differently if not understood at 100% - and for detecting hypomania, nuances can sometimes be very important), we preferred to develop a Tunisian Arabic version. Thus, the HCL - 32 was translated into the Tunisian Arabic language by a bilingual native editor, then back-translated into English by another bilingual native editor. A third, independent researcher, with a deep knowledge of the tool, contributed to harmonize the translation and back-translation of the HCL - 32. Potential issues in reading or unclear items were addressed in a pilot study with eight patients, whose help served to complete the translation of the HCL - 32 in its final form.

The HCL - 32 is a self-report questionnaire comprising a list of 32 possible hypomanic symptoms, to be rated as present or absent in a yes/no format. Additional questions concern the duration of the hypomanic experience and the impact on the family, social, and work life. A total score is yielded by the sum of all “yes” replies. A cut-off of 14 out of 32 items is optimal, in terms of sensitivity and specificity, for identifying BD [25].

### Statistics

Data were imputed in Excel, then they were coded and analyzed using the Statistical Package for Social Sciences (SPSS) version 27. Specific analyses were done with dedicated packages running [38] in R. All tests were two-tailed, with alpha set at *p* < 0.05.

Descriptive statistics were reported as means with standard deviation, or as counts and percentages. Non-parametric tests were used to assess differences between groups or correlations among variables, except for age.

To assess the usability of the scale in the target population, we calculated floor and ceiling effects [39]. They occur when more than 15% of respondents score at the minimum (in this case, zero) or the maximum scores (either 13 for the MDQ or 32 for the HCL-32). The occurrence of floor or ceiling effects indicates that extreme items are missing in the lower or upper end of the scale, indicating limited content validity.

Reliability was measured as internal coherence using Cronbach’s alpha. The Bayesian reliability analysis, as implemented in JASP 0.14.1 version [40], has been used to calculate the Cronbach’s alpha. According to a shared rule-of-thumb, Cronbach’s alpha is considered “moderate” when it is > 0.6 and “good” when it is > 0.7 [41].

Before testing the criterion validity of the MDQ and the HCL-32, confirmatory factor analysis (CFA) was applied to the items of both questionnaires to make sure that a single global score was an appropriate summary measure of the screeners in the total sample. Preliminary analysis with the Mardia’s test [42] revealed a violation of multivariate normality in the data for both the MDQ and the HCL-32 (skew’s *p* < 0.0001 in both analyses). Therefore, the Diagonally Weighted Least Squares (DWLS) estimator was used in CFA. To assess goodness of fit estimation, we used the following parameters: the chi-square, the Comparative Fit Index (CFI), the Root Mean Square Error of Approximation (RMSEA), and the Standardized Root Mean Square Residual (SRMR). In the presence of a chi-square with *p* < 0.001, as expected with large samples (*n* > 300), RMSEA values of 0.08 or lower, SRMR values of 0.09 or lower, and CFI values of 0.90 or higher were considered an indication of acceptable fit according to conventional rules of thumb [43]. The following model were tested: an unidimensional model, which assumes all core items of the MDQ or the HCL-32 tap into a single dimension of propensity to the manic/hypomanic syndrome; a two-factor model of elated and irritable dimensions, as in Ouali et al., 2020 for the MDQ [35] and in Meyer et al., 2007 for the HCL-32 [15]; and these two-factor models’ bifactor implementation [44], which assumes that most variance in the scores is attributable to a general factor resulting from the loading of all items on a single dimension of propensity to the manic/hypomanic syndrome, with an additional but residual variance purportedly explained by the loading of the items on the “elated” and the “irritable” dimensions, as defined above. To check for reasonable unidimensionality of the general factor extracted from the bifactor model, the explained common variance (ECV), the percentage of uncontaminated correlations (PUC), and the Omega Hierarchical (ωH) were calculated [45]. We also calculated the construct replicability H index of Hancock and Mueller (2001) [46]. H values of .80 or higher indicate a well-defined latent variable, which is more likely to be stable across studies. The presence of multidimensionality might be discarded when ECV is higher than .60 and ωH > .70 or PUC > .70 [45]. CFA models were tested with the “lavaan” package running in R [47].The calculation of the bifactor indices was done with the “Bifactor Indices Calculator” package running in R [48].

The receiver operating characteristics (ROC) curve was used to test for the criterion validity of the tools. Criterion validity was intended the degree to which the scores of the instrument were an adequate reflection of a “gold standard” [49]. For the purposes of this study, we used the diagnosis assigned after the SCID interview as a “gold standard” for reference. Thus, the ROC curve analysis was used to distinguish between diagnostic groups for both the MDQ and the HCL-32. Sensitivity was defined as the probability of a true positive case, i.e. the probability of identifying a patient with BD. Specificity was the probability of a true negative case, i.e. the probability of identifying a patient without BD. We also derived the positive predictive value (PPV), i.e., the probability that a person is a case of BD when a positive test result is observed; the negative predictive value (NPV), i.e., the probability that a person is not a case of BD when a negative test result is observed; and the positive diagnostic likelihood ratio, which is the odds ratio that a positive test will be observed in a population of people with BD compared to the odds that the same result will be observed among a population of people without BD. The accuracy in the prediction was estimated from the area under the curve (AUC; with 95% confidence interval). Agreed threshold for the AUC were: ≤ .70, poor; between .70 and .80, fair; between .80 and .90, good; above .90, excellent [50].

We used the “pROC” package running in R to perform the ROC analysis [51], while the best cut-off point for the MDQ and the HCL-32 was established according to the Youden (1950) method with the “Optimal Cut points” package [52]. The comparison of the two paired ROC curves for MDQ and HCL-32 in the same sample was done with a bootstrap test according to Hanley and McNeil (1983). The test was performed with the “pROC” package.

#### Sample size estimation and power analysis

CFA and ROC analysis impose some requirements for sample size. As for the CFA, with DWLS applied to binary or ordinal data, a sample size between 200 and 500 subjects is enough for model convergence and parameters’ estimation, according to Monte Carlo simulation studies (Bandalos, 2014). Thus, the global sample size in this study was sufficient to conduct CFA.

As for the ROC analysis, with alpha set at 0.05 and power at 80% (beta = 0.20), with 59 cases of BD and 281 controls, we could detect an AUC as low as 0.612, which is even lower than the minimum fair AUC (0.700). With the same parameters and 59 cases of BD and 86 cases of MDD, we could test the diagnostic ability of the screeners in discriminating the two diagnoses detecting an AUC as low as 0.632. This power analysis was performed with the “pROC” package running in R [53].

## Results

The sample included 86 patients diagnosed with MDD, 22 patients diagnosed with BD-I and 37 patients diagnosed with BD-II. There were also 281 putatively healthy controls (Table 1).

**Table 1:** General characteristics of the participants included in the study

	MDD	BD-I	BD-II	Healthy Controls	
	*N* = 86	*N* = 22	*N* = 37	*N* = 281	
Sex					χ^2^ = 4.52; df = 3; *p* = 0.21
Men	30 (35%)	13 (59%)	16 (43%)	110 (39%)	
Women	56 (65%)	9 (41%)	21 (57%)	171 (61%)	
Age	42 (11)	42 (9)	41 (9)	38 (13)	F[3;421] = 2.85; *p* = 0.037
Education					χ^2^ = 12.03; df = 9; p = 0.21
College or University	39 (45%)	8 (36%)	17 (46%)	102 (55%)	
Age of onset of psychopathology	35 (12)	35 (10)	31 (10)	–	F[2;142] = 1.34; *p* = 0.26
Family history of depression	26 (31%)	6 (27%)	21 (57%)	–	χ^2^ = 8.40; df = 2; *p* = 0.015
Family history of bipolar disorder	4 (5%)	6 (27%)	9 (24%)	–	χ^2^ = 12.9; df = 2; *p* = 0.002
Suicide attempt	14 (16%)	12 (54%)	11 (30%)	–	χ^2^ = 14.03; df = 2; *p* = 0.001
Admission to psychiatric services	16 (18%)	11 (50%)	10 (27%)	–	χ^2^ = 9.14; df = 2; *p* = 0.010
Received a prescription of antidepressants	70 (84%)	19 (86%)	33 (89%)	–	χ^2^ = 0.73; df = 2; *p* = 0.69
Received a prescription of a second-generation antipsychotic	9 (10%)	3 (13%)	4 (11%)		Freeman-Halton extension of Fisher’s exact test: *p* = 0.93
Received a prescription of lithium	0 (0%)	2 (9%)	0 (0%)	–	Freeman-Halton extension of Fisher’s exact test: *p* = 0.026

Data were expressed as counts and percentages within brackets or as mean and standard deviation within brackets

There were no differences by gender or maximum education level among participants; controls were marginally younger than the patients (partial eta-squared = 0.020).

Clinical data were available for patients only. There was no relevant difference in the age of onset of the psychopathology among groups. A family history of depression was observed more often in patients diagnosed with BD-II, while a family history of BD was observed in just 5% of patients diagnosed with MDD and in about 25% of those diagnosed with BD (Table 1 for details).

Patients diagnosed with BD-I were more likely to have attempted suicide and have been more often admitted to a psychiatric service than patients with MDD or BD-II. A prescription of an antidepressant was received by most patients, with no differences by diagnosis. A second-generation antipsychotic was prescribed in about 10% of cases, again with no difference by diagnosis. Lithium was rarely prescribed and only in patients diagnosed with BD-I.

Overall, 86 patients with MDD, 58 patients with BD (either BD-I or BD-II), and 265 controls completed the MDQ; while the HCL-32 was completed by 64 patients with MDD, 32 with BD, and 225 controls.

### Floor or ceiling effects

There were no floor effects for the MDQ: 25 controls (8.9%) and just 1 with MDD (1%) scored zero on the MDQ (χ^2^ = 11.85; df = 2; *p* = 0.003). However, a modest ceiling effect was observed for the MDQ: 4 controls (1.4%) and 11 patients with BD (17.7%) scored 13 on the MDQ (χ^2^ = 44.38; df = 2; *p* < 0.0001).

There were no floor and ceiling effects for the HCL-32. Overall, in the sample 7 patients scored zero on the HCL-32: 5 controls, 2 with MDD, none with BD (χ^2^ = 1.28; df = 2; *p* = 0.52). No participants scored 32 on the HCL-32.

### Reliability of the questionnaires

Cronbach’s alpha for MDQ was 0.79 (95%CI: 0.76–0.83) in controls; 0.78 (0.75–0.82) in patients with MDD; and 0.71 (0.60–0.81) in patients diagnosed with BD. Cronbach’s alpha for HCL-32 was, respectively, 0.85 (0.82–0.87) in controls, 0.80 (0.74–0.85) in MDD, and 0.76 (0.68–0.85) in BD.

### Confirmatory factor analysis of the factor structure of the MDQ and the HCL-32

For both the MDQ and the HCL-32, the bifactor implementation of the two-factor model had the best fit according to the predefined parameters (Table 2).

**Table 2:** Confirmatory factor analysis of the MDQ and the HCL-32. Goodness-of-fit indices of the tested models

Model	χ^2^	df	p	CFI	RMSEA (90%CI)	SRMR
*MDQ*						
Unidimensional	329.2	65	< 0.0001	0.894	0.100 (0.089–0.111)	0.089
Two-factor	213.8	53	< 0.0001	0.924	0.086 (0.074–0.098)	0.045
Bifactor	77.3	53	0.016	0.990	0.034 (0.015–0.049)	0.045
*HCL-32*						
Unidimensional	1864.6	464	< 0.0001	0.798	0.097 (0.093–0.102)	0.109
Two-factor	1562.0	463	< 0.0001	0.842	0.086 (0.081–0.091)	0.098
Bifactor	715.8	432	< 0.0001	0.959	0.045 (0.039–0.051)	0.067
*Threshold for fit*			*p* > 0.05	0.90	≥0.08	≥0.09

For the bifactor model of the MDQ, H = 0.79, ECV = 0.54, PUC = 0.60, and ωH = 0.64.

For the bifactor model of the HCL-32, H = 0.80, ECV = 0.33, PUC = 0.48, and ωH = 0.37.

Thus, for both the MDQ and the HCL-32 there is some indication in favor of a single, reproducible latent component. However, the multidimensionality in the data might influence the results that can be derived from a global summary score.

### Discriminant capacity of the MDQ and the HCL-32

Patients diagnosed with BD scored higher than patients diagnosed with MDD and controls on both the MDQ and the HCL-32 (Table 3).

**Table 3:** Scores of the HCL-32 and the MDQ by subgroup of participants

	MDD	BD	Healthy Controls	Kruskal-Wallis	
	*N* = 86	*N* = 58	*N* = 265		
MDQ	5.3 (2.3)	10.0 (2.5)	5.7 (3.4)	H = 77.9; df = 2; *p* < 0.0001	∑^2^ = 0.191
	*N* = 64	*n* = 32	*N* = 225		
HCL-32	11.9 (5.8)	19.5 (5.3)	14.5 (6.1)	H = 31.4; df = 2; *p* < 0.0001	∑^2^ = 0.098

Data were expressed as mean and standard deviation within brackets

According to the epsilon-squared effect size (Tomczak and Tomczak, 2014), about 20% of the variance in the sample was attributable to the differences in MDQ by groups, and 10% was attributable to the differences in HCL-32 by groups.

### ROC analysis

**Fig. 1:**
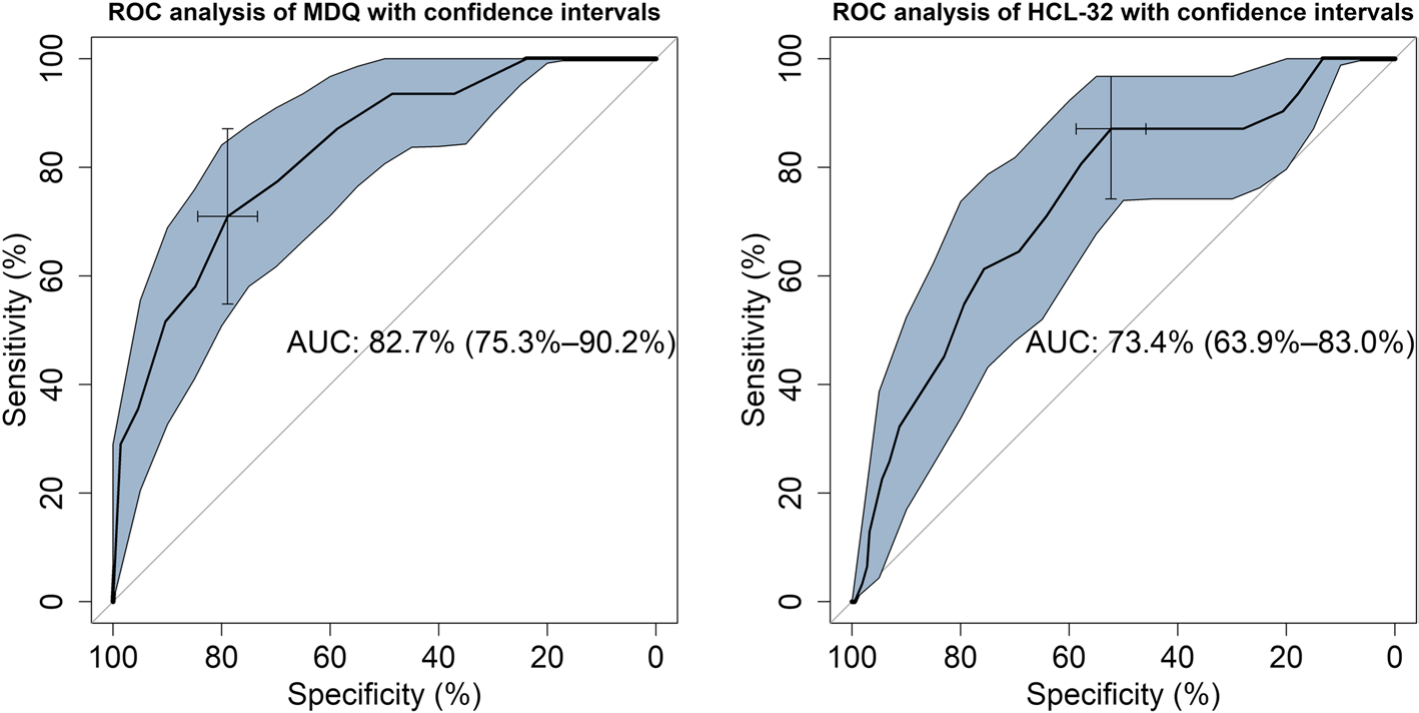
Receiver operator characteristic (ROC) curve of the predictive capacity of the Tunisian MDQ (on the left) and the Tunisian arabic HCL-32 (on the right) in differentiating patients with BD from healthy controls. Sensitivity and specificity are reported as percentages, with a cross indicating on the curve the best compromise between them (corresponding to the cut-off). The area under the ROC curve (AUC) is reported alongside its 95% confidence interval

The MDQ and the HCL-32 were able to distinguish patients diagnosed with BD from putatively healthy controls, with better AUC in MDQ (82.7; 95%CI: 75.3–90.2) than in HCL-32 (73.4; 63.9–83.0) (Fig. 1).

**Fig. 2:**
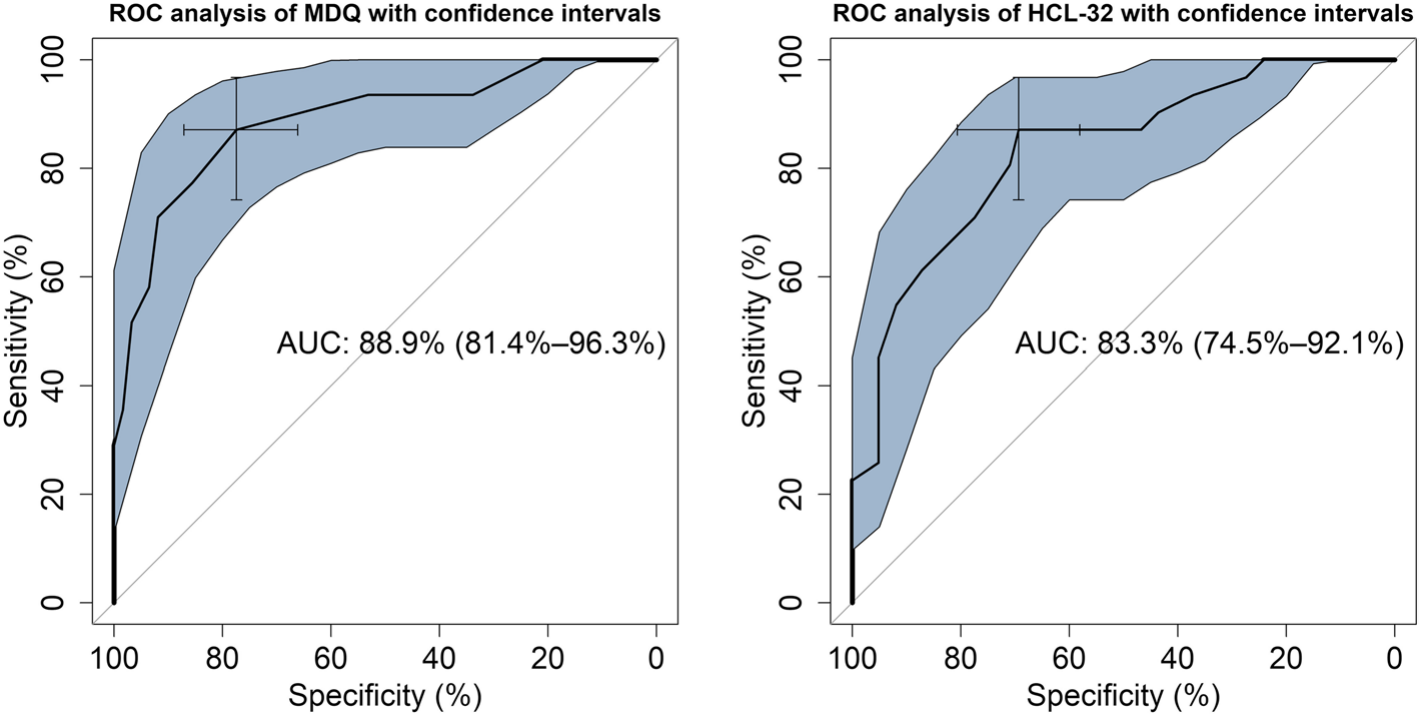
Receiver operator characteristic (ROC) curve of the predictive capacity of the Tunisian MDQ (on the left) and the Tunisian arabic HCL-32 (on the right) in differentiating patients with BD from patients with MDD. Sensitivity and specificity are reported as percentages, with a cross indicating on the curve the best compromise between them (corresponding to the cut-off). The area under the ROC curve (AUC) is reported alongside its 95% confidence interval

The MDQ (AUC: 88.9; 81.4–96.3) and the HCL-32 (AUC: 83.3; 74.5–92.1) were equally able to distinguish patients diagnosed with BD from patients with MDD (Fig. 2).

**Fig. 3:**
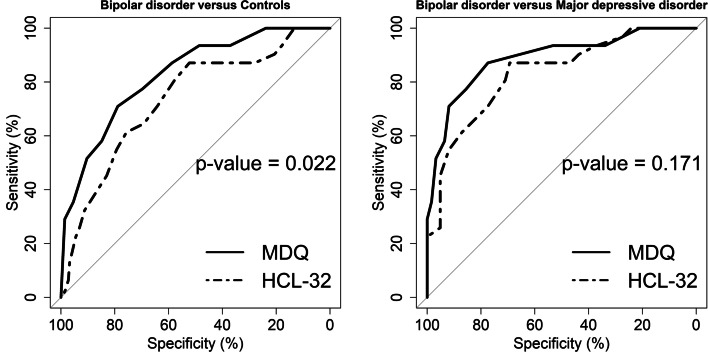
Comparison with the Hanley and McNeil’s test between the Tunisian arabic MDQ and the Tunisian arabic HCL-32 in distinguishing patients with BD from putatively healthy controls (on the left), or from patients with MDD (on the right)

When compared with the Hanley and McNeil’s test, the MDQ was confirmed better than the HCL-32 in distinguishing patients with BD from putatively healthy controls, while no difference was found between the two screeners in the differentiation of patients with BD from those with MDD (Fig. 3).

The best threshold for the differentiation of patients with BD from patients with MDD was 7 for the MDQ (Fig. A1) and 15 for the HCL-32 (Fig. A2).

Sensitivity and specificity at the best threshold were 87 and 77%, respectively, for the MDQ, and 87 and 69% for the HCL-32. Both screeners had a better NPV (92.3 and 91.4%, respectively) than PPV (65.8 and 58.7%). The positive diagnostic likelihood ratio was modestly higher for the MDQ (3.86) than for the HCL-32 (2.84).

In the investigated samples, 109 controls (41.1%), 21 patients with MDD (24.4%), and 52 patients with BD (89.7%) scored at or above the cut-off on the MDQ (χ^2^ = 63.14; df = 2; *p* < 0.0001). The corresponding figures for the HCL-32 were 108 (48%) among controls, 21 (32.8%) among patients with MDD, and 28 (87.5%) among patients with BD (χ^2^ = 25.78; df = 2; p < 0.0001).

## Discussion

In this study, both the MDQ and the HCL-32 were able to distinguish patients diagnosed with BD from patients diagnosed with MDD, with a good accuracy (when measured with AUC) and an informative positive diagnostic likelihood ratio (above 2). Both screeners were more able to exclude the presence of a BD than to confirm it, on the basis of their PPV and NPV. Reliability was good for both the MDQ and the HCL-32. In controls, too, the reliability of the two screeners was good to excellent.

The controls were probably likely to admit socially acceptable hyperthymic traits, such as being more sociable than their peers or being exuberant in social circumstances. This might explain the higher fraction of controls than of MDD patients scoring at or above the cut-off for screening a BD. However, the reporting of hypomanic-like symptoms by controls does not necessarily correspond to real, true episodes of hypomania. Moreover, the higher reporting of hyperthymic traits and hypomanic-like symptoms by controls was not corroborated by an independent source.

This is the first study to have tested a bifactor structure of the MDQ and the HCL-32. In past investigations, a two-factor structure was repeatedly reported to explain the distribution of the scores of the two screeners, with some items reflecting a propensity to elated behaviors, and another set of items being a reflection of an impulsive/irritable mood [24, 32, 54, 55]. In this study, this two-factor solution did not show a good fit according to the predefined parameters. The bifactor implementation of this two-factor model, instead, showed a good fit to the data. The excessive reliance on the exploratory factor analysis over the confirmatory factor analysis of past studies might in part explain the difference between this and previous investigations of the topic. It should be noted that both the MDQ and the HCL-32 are usually applied as a single factor screener, thus a bifactor model of a multidimensional structure of the screeners is the best approximation to the expected factor structure of the tools and to its current use. It should be noted that in this study, the indicators of the appropriateness of the general factor of the bifactor model were below the accepted threshold for full acceptance of the general factor as a single summary score of the tools. This may depend on the application of the model to a sample that included both patients and putatively healthy controls. This might have inflated the impact of the multidimensionality of both tools, since the elated and impulsive/irritable experience of the patients might be qualitatively different from the corresponding experience in people without a mood disorder.

In this sample, the best cut-off for the HCL-32 was close to the one reported in past studies that were carried out in the Western samples, usually about 14 or 15. However, in some non-Western samples, such as in the Arabian study of Fornaro et al. (2015) [34] or the Brazilian sample of patients of Soares et al. (2010), higher cut-offs were reported, around 17/18. Fornaro et al. (2015) included inpatients, while Soares et al. (2010) [18] enrolled outpatients. Probably both severity and cultural differences in admitting some hypomanic symptoms might have had a role in explaining the higher cut-offs in those studies. In this study, the sensitivity and specificity of the HCL-32 in discriminating patients with BD from those with MDD were, respectively, .87 and .69, somehow higher than the corresponding figures in the Soares et al. study (.75 and .58), and close to the values observed by Perugi et al. (2012) [56] in their large Italian study (.85 and .78). Fornaro et al. [34] found similar values of sensitivity (.82) and specificity (.77) of their version of the HCL-32 in the discrimination of Arabic patients with MDD from those with BD. Both the Perugi et al. (2012) study and Fornero et al. [34] study found a higher specificity of the applied version of the HCL-32, suggesting that sample composition might affect the detection of hypomanic symptoms. Indeed, in the present study, we enrolled a larger fraction of patients with BD-II than with BD-I, while the Fornero et al. [34] study had a ratio of BD-I to BD-II = 4.7. This might be considered a limitation of the present study, but in community samples, the lifetime prevalence of BD-II tends to be higher (1.57%; 95%CI: 1.15–1.99) than the lifetime prevalence of BD-I (1.06%; 0.81–1.31) [26]. Moreover, in past studies, patients had already received a diagnosis of BD, thus might have been more prone to admit hypomanic symptoms.

Overall, the two screeners revealed ease of use, albeit requiring some degree of literacy. Time to fill in was in general minimal for patients with adequate reading skills, but sometimes it requires more time in older patients. Nevertheless, both the MDQ and the HCL-32 might represent valuable help in busy primary care settings, favoring the recognition of cases in need of closer evaluation.

### Strengths and limitations

The major strength of the study is its design, which was as close as possible to clinical reality, as we included patients only complaining of depressive signs and symptoms, but did not have any precompiled diagnosis of unipolar or bipolar depression when they first presented. This is a major difference from most of the other studies about MDQ and HCL-32, which often included patients that had already received a diagnosis of BD [1, 34], and might have received some clue about the symptoms they are expected to admit [57]. Several limitations have to be taken into account. Some of the questionnaires, either MDQ or HCL-32, were incomplete, especially among patients with BD. This depended mainly on patients leaving blank some items, such as item 6 (about wanting to travel) or 7 (about risky driving) of the HCL-32 because they do not habitually do the enquired action (they do not travel or drive a car), thus they didn’t know how to reply to the question. As a consequence, we had to discard some of the cases and this resulted in a loss of power for the analysis. In particular, we had not enough cases with BD-II to test the discriminant capacity of the tools with respect to MDD, the main usage of a screening tool to identify BD. Indeed, while manic episodes are more likely to be recognized by clinicians and to be remembered by the patients, the hypomanic episodes are precisely those that complicate the diagnosis of BD in the clinical setting.

## Conclusion

Despite its limitations, this study showed the good capacity of both the MDQ and the HCL-32 as screening tools to be used to differentiate patients with BD from patients with MDD. Both screeners work best in excluding the presence of BD in patients with MDD, which is an advantage in deciding whether or not to prescribe an antidepressant, which can have known negative effects in patients with BD [58]. When the screener is positive for the presence of BD, it may prompt a deeper investigation of past manic/hypomanic episodes that might have been overlooked at the first assessment.

## Supplementary Information

**Additional file 1.** Operating characteristics of the Tunisian arabic MDQ for various threshold scores among patients diagnosed with a current episode of major depressive disorder either in the course of a unipolar or bipolar mood disorder as diagnosed with the SCID. Specificity and sensitivity are plotted per percentage of subjects and the number of items checked positive on the screener.

**Additional file 2.** Operating characteristics of the Tunisian arabic HCL-32 for various threshold scores among patients diagnosed with a current episode of major depressive disorder either in the course of a unipolar or bipolar mood disorder as diagnosed with the SCID. Specificity and sensitivity are plotted per percentage of subjects and the number of items checked positive on the screener.

**Additional file 3.**

## Data Availability

The dataset of this study is available from the corresponding author on reasonable request.
