# Supplementary figures and images for: Accuracy of the Arabic HCL - 32 and MDQ in detecting patients with bipolar disorder
Source: BMC Psychiatry. 2023 Jan 26;23:70. doi: 10.1186/s12888-023-04529-x (PMC9878752; doi:10.1186/s12888-023-04529-x)

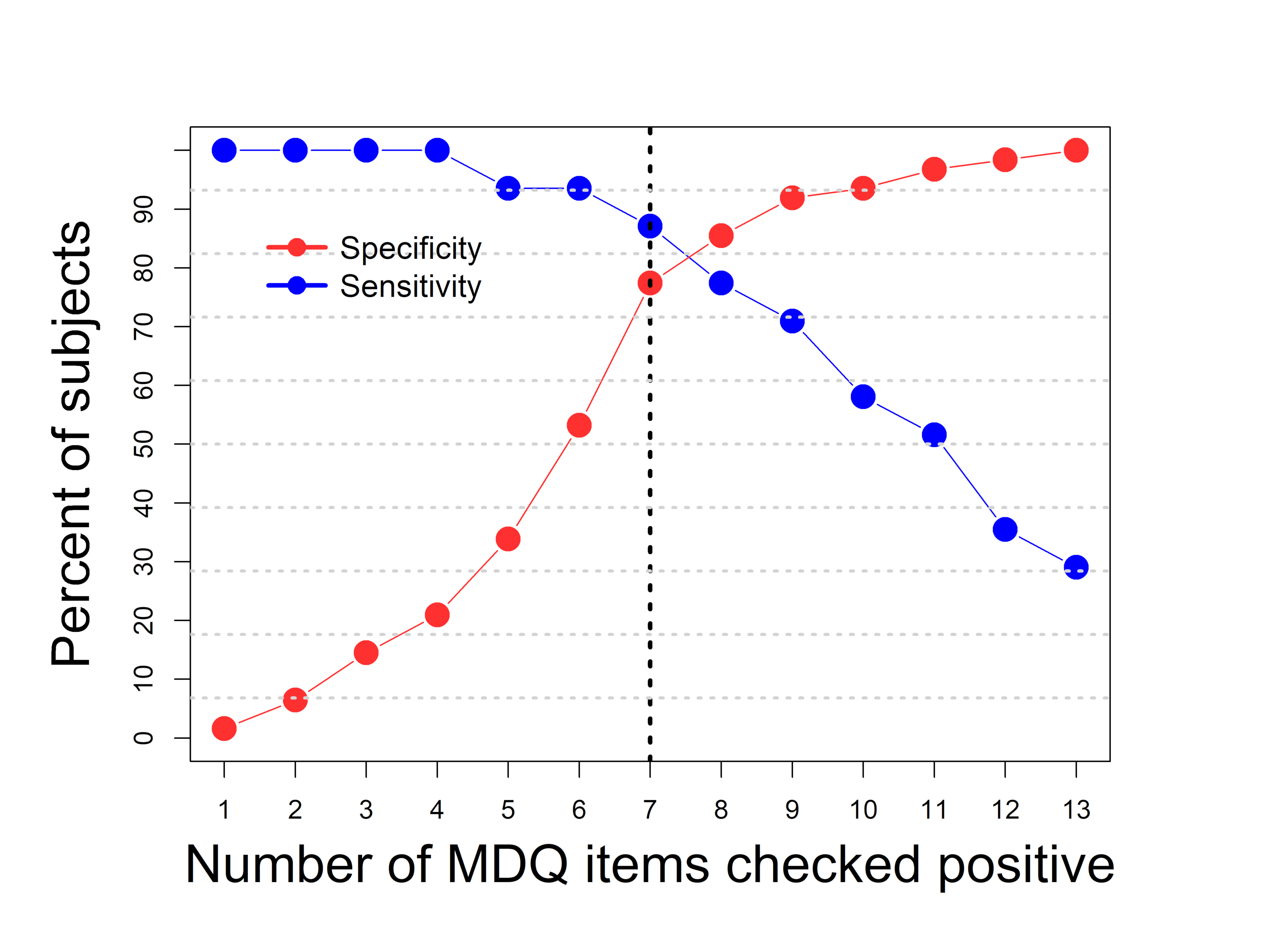

Supplement: Supplementary file 1 — Additional file 1. Operating characteristics of the Tunisian arabic MDQ for various threshold scores among patients diagnosed with a current episode of major depressive disorder either in the course of a unipolar or bipolar mood disorder as diagnosed with the SCID. Specificity and sensitivity are plotted per percentage of subjects and the number of items checked positive on the screener. [file 12888_2023_4529_MOESM1_ESM.jpg]

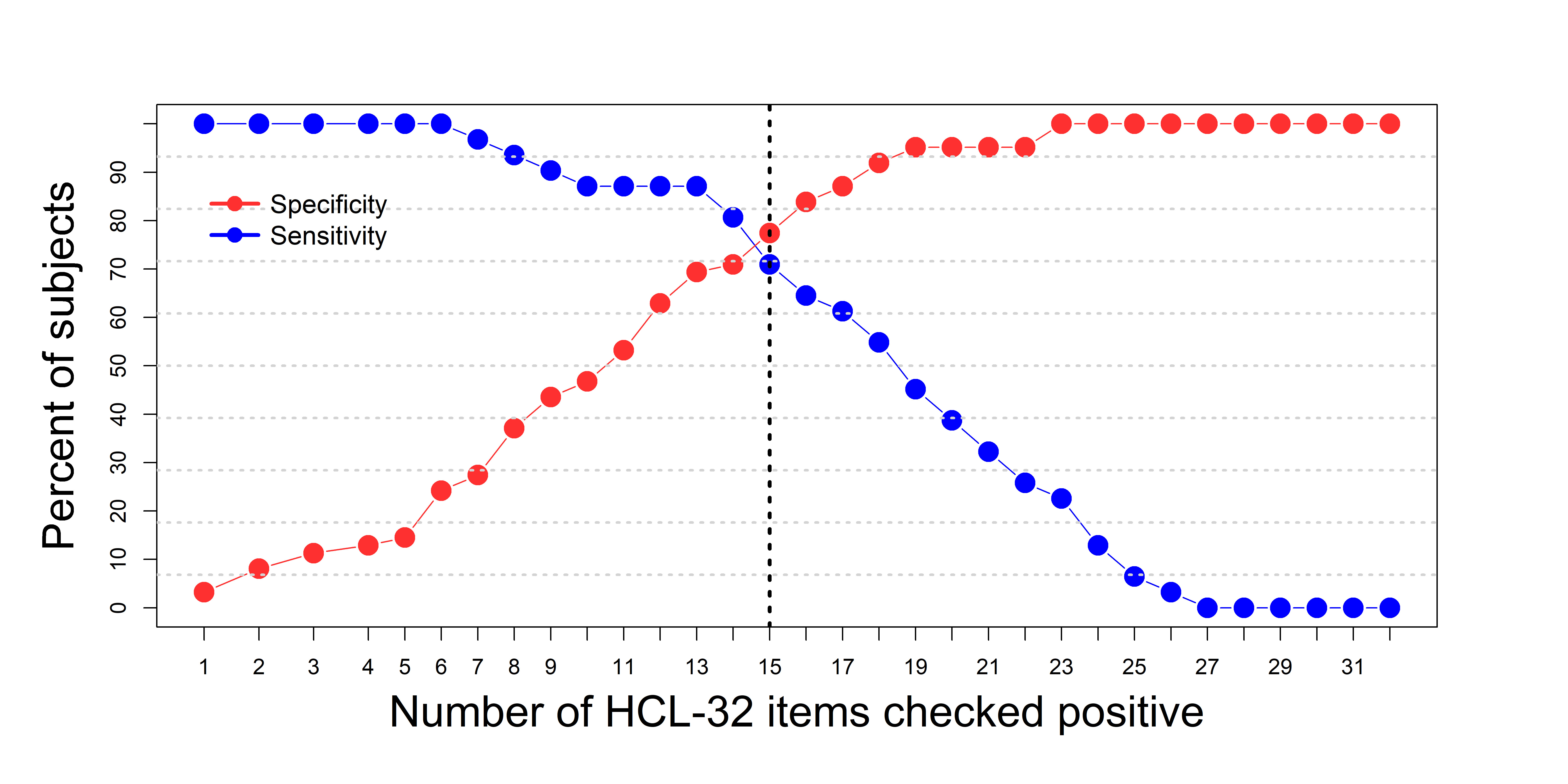

Supplement: Supplementary file 2 — Additional file 2. Operating characteristics of the Tunisian arabic HCL-32 for various threshold scores among patients diagnosed with a current episode of major depressive disorder either in the course of a unipolar or bipolar mood disorder as diagnosed with the SCID. Specificity and sensitivity are plotted per percentage of subjects and the number of items checked positive on the screener. [file 12888_2023_4529_MOESM2_ESM.jpg]
