# Supplementary material for: Accuracy of the Arabic HCL - 32 and MDQ in detecting patients with bipolar disorder
Source: BMC Psychiatry. 2023 Jan 26;23:70. doi: 10.1186/s12888-023-04529-x (PMC9878752; doi:10.1186/s12888-023-04529-x)
Supplement: Supplementary file 3 — Additional file 3. [file 12888_2023_4529_MOESM3_ESM.docx]

HCL-32 Items

| NO | Yes | ITEMS |  |
| --- | --- | --- | --- |
| 🞏 | 🞏 | I need less sleep  نحتاج لاقل نوم من العادة | .1 |
| 🞏 | 🞏 | I feel more energetic and more active  نحس بروحي أنشـط و عندي طاقة أكثر من العادة | .2 |
| 🞏 | 🞏 | I am more self-confident  نحس الي عندي ثقـة أكثـر في روحي | .3 |
| 🞏 | 🞏 | I enjoy my work more  نعمل كيف على خدمتي أكثـر | .4 |
| 🞏 | 🞏 | I am more sociable (make more phone calls, go out more  نحب نخالط العباد أكثـر (نتكلم أكثر في التليفون و نخرج أكثـر) | .5 |
| 🞏 | 🞏 | I want to travel and/or do travel more  نحب نسافـر ، و انسافـر أكثـر | .6 |
| 🞏 | 🞏 | I tend to drive faster or take more risks when driving  نجري و نخاطـر أكثـر في السـوقـان | .7 |
| 🞏 | 🞏 | I spend more money/too much money  نصـرف ياسر، أكثـرمن العادة | .8 |
| 🞏 | 🞏 | I take more risks in my daily life (in my work and/or other activities)  نخاطر أكثـر في حيـاتي اليومية (في خدمتي و في حاجات أخرى ) | .9 |
| 🞏 | 🞏 | I am physically more active (sport etc.)  نتحرّك أكثر (الرياضـة) | .10 |
| 🞏 | 🞏 | I plan more activities or projects  نعمـل أكثـر برامج و مشاريع | .11 |
| 🞏 | 🞏 | I have more ideas, I am more creative  تكثر أفكاري ، وابـداعاتي | .12 |
| 🞏 | 🞏 | I am less shy or inhibited  تنقص علي الحشمـة (وكاني ماعادش عندي فرينوات) | .13 |
| 🞏 | 🞏 | I wear more colorful and more extravagant clothes/make-up  نلبس حوايج ملوٌنين أكثر/ مشعشعين / نمكيج أكثر | .14 |
| 🞏 | 🞏 | I want to meet or actually do meet more people  نحب نقابل أكثر نـاس/ أووليت نقابل أكثر عباد | .15 |
| 🞏 | 🞏 | I am more interested in sex,  نهتم أكثربالجنس، وعندي أكثر رغبة جنسية | .16 |
| 🞏 | 🞏 | I am more flirtatious and/or am more sexually active  نشكل أكثر، و نمارس أكثر الجنس | .17 |
| 🞏 | 🞏 | I talk more  نتكلم أكثرمن العادة | .18 |
| 🞏 | 🞏 | I think faster  نفكّر أسرع من العادة | .19 |
| 🞏 | 🞏 | I make more jokes or puns when I am talking  نفدلك وننكت أكثر من العادة | .20 |
| 🞏 | 🞏 | I am more easily distracted  مخي يسهى (يسرح) بسهولة أكثر | .21 |
| 🞏 | 🞏 | I engage in lots of new things  نعمل برشة حاجات جدد | .22 |
| 🞏 | 🞏 | My thoughts jump from topic to topic  أفكاري تنقز من حاجة لحاجة | .23 |
| 🞏 | 🞏 | I do things more quickly and/or more easily.  نعمل ا لحاجات بأكثر سرعة و أكثر سهولة | .24 |
| 🞏 | 🞏 | I am more impatient and/or get irritable more easily  ما عادش عندي وسع بال ونتنرفزفيسع | .25 |
| 🞏 | 🞏 | I can be exhausting or irritating for others  نجّم انتعّب و نّرفز العباد أكثر | .26 |
| 🞏 | 🞏 | I get into more quarrels  نتدخل في برشة مشاكل / عرك | .27 |
| 🞏 | 🞏 | My mood is higher, more optimistic  نحس روحي شايخ / زاهى/ متفائل أكثر | .28 |
| 🞏 | 🞏 | I drink more coffee  نشرب أكثر قهاوي | .29 |
| 🞏 | 🞏 | I smoke more cigarettes  نتكيف أكثرسواقر | .30 |
| 🞏 | 🞏 | I drink more alcohol  نشرب أكثرمشروبات كحولية | .31 |
| 🞏 | 🞏 | I take more drugs (sedatives, anxiolytics, stimulants…)  نستهلك أكثر دوايات (مسكنات ،مهدئات، منشط) | .32 |
